# Multidrug Resistance in *Escherichia coli* Strains Isolated from Infections in Dogs and Cats in Poland (2007–2013)

**DOI:** 10.1155/2015/408205

**Published:** 2015-01-15

**Authors:** Magdalena Rzewuska, Michał Czopowicz, Magdalena Kizerwetter-Świda, Dorota Chrobak, Borys Błaszczak, Marian Binek

**Affiliations:** ^1^Department of Preclinical Sciences, Faculty of Veterinary Medicine, Warsaw University of Life Sciences, Ulica Ciszewskiego 8, 02-786 Warsaw, Poland; ^2^Laboratory of Veterinary Epidemiology and Economics, Faculty of Veterinary Medicine, Warsaw University of Life Sciences, Ulica Nowoursynowska 159c, 02-776 Warsaw, Poland; ^3^Department of Pathology and Veterinary Diagnostics, Faculty of Veterinary Medicine, Warsaw University of Life Sciences, Ulica Nowoursynowska 159c, 02-776 Warsaw, Poland

## Abstract

The antimicrobial susceptibility of *Escherichia coli* isolates associated with various types of infections in dogs and cats was determined. The studied isolates were most frequently susceptible to fluoroquinolones and the extended-spectrum cephalosporins (ESCs), antimicrobials commonly used in treatment of infections in companion animals. However, an increase in the percentage of strains resistant to *β*-lactam antibiotics including ESCs was noted between January 2007 and December 2013. The frequency of multidrug-resistant (MDR) *E. coli* isolation (66.8% of isolates) is alarming. Moreover, the statistically significant increase of the percentage of MDR isolates was observed during the study period. No difference in the prevalence of multidrug resistance was found between bacteria causing intestinal and extraintestinal infections and between canine and feline isolates. Nonhemolytic *E. coli* isolates were MDR more often than hemolytic ones. Our study showed the companion animals in Poland as an important reservoir of MDR bacteria. These results indicate that continuous monitoring of canine and feline *E. coli* antimicrobial susceptibility is required. Furthermore, introduction and application of recommendations for appropriate use of antimicrobials in small animal practice should be essential to minimize the emergence of multidrug resistance among *E. coli* in companion animals.

## 1. Introduction

The antimicrobial resistance of bacterial pathogens of human and animal origin has been increasing worldwide since antibiotics became used in the 1940s. The emergence and dissemination of multidrug-resistant (MDR) strains of various bacterial species pose serious challenges for effective medical treatment. The occurrence of MDR bacteria is associated with the extensive use of broad-spectrum antimicrobials in treating human and animal infections [1, 2]. Antimicrobials commonly used in small animal veterinary practice are *β*-lactams and fluoroquinolones.

An occurrence of antimicrobial resistant strains in food-producing and companion animals, which share the same environment and remain in close contact with people, has been demonstrated for various zoonotic pathogens, such as* Salmonella* spp.,* Staphylococcus aureus,* or* Campylobacter* spp. [3–5]. MDR bacteria have been isolated from many animal species including pigs, cattle, chickens, turkeys, dogs, cats, and rodents [6–8]. The possibility of transmission of these bacteria, including pathogenic* Escherichia coli*, between companion animals and humans has been documented [9, 10].

*E. coli* is among the most important canine and feline bacterial pathogens associated with extraintestinal infections including those of the urinary, respiratory, and reproductive tracts [11]. This bacterium can also cause gastrointestinal tract infections [11]. Selection of appropriate antimicrobials is crucial for effective therapy of these infections and substantially decreases the risk of development of multidrug resistance in these pathogenic or commensal bacteria. The antimicrobial resistance of canine and feline* E. coli* has been reported worldwide, including some European [1, 9, 12–14] and Asian [7] countries, the USA [6], Australia [15], and Brazil [16]. However, as far as the authors know, no data have been published yet regarding antimicrobial resistance profiles of* E. coli* isolates from dogs and cats in Poland.

This study was conducted to investigate and analyze the antimicrobial resistance of* E. coli* isolates from diseased dogs and cats, with a particular emphasis on multidrug resistance.

## 2. Materials and Methods

### 2.1. Bacterial Isolates

All* E. coli* isolates (*n* = 730) investigated in this study were obtained from clinical specimens submitted to the Diagnostic Laboratory of the Division of Microbiology, Faculty of Veterinary Medicine at the Warsaw University of Life Sciences (Poland), from January 2007 to December 2013. Clinical samples were collected from dogs and cats with various types of infections (Table 1).* E. coli* isolates were cultured and identified using standard microbiological diagnostic techniques including the API 20 *E* test (bioMérieux, France). Based on the presence or absence of *β*-haemolysis on blood agar, the isolates were classified as haemolytic or nonhaemolytic, respectively. Based on clinical and microbiological diagnosis, all the* E. coli* isolates included in the study were recognized as the sole agent involved in the infection. In case of fecal samples, a parasitological examination was additionally performed to exclude parasitic disease. A reference strain,* E. coli* ATCC 25922, was used as a quality control in the susceptibility tests.

### 2.2. Determination of Antimicrobial Susceptibility

Antimicrobial susceptibility testing was performed by the agar disk diffusion method according to the Clinical and Laboratory Standards Institute (CLSI) guidelines [17]. Briefly, the density of bacterial inoculum (a saline suspension) used in the test was equivalent to a 0.5 McFarland standard. Bacteria were spread to a Mueller-Hinton agar plate and the antimicrobial agent disks were placed on the surface of the medium. The plates were incubated for 18 hours at 35°C in aerobic conditions, and then the diameters of complete growth inhibition zones were measured. Susceptibility to 13 antimicrobials was determined: amoxicillin (AMX: *n* = 726), amoxicillin/clavulanic acid (AMC: *n* = 724), cefuroxime (CXM: *n* = 410), cefotaxime (CTX: *n* = 388), cefovecin (VEC: *n* = 450), gentamicin (GEN: *n* = 720), neomycin (NEO: *n* = 724), streptomycin (STR: *n* = 724), norfloxacin (NOR: *n* = 269), enrofloxacin (ENR: *n* = 112), marbofloxacin (MAR: *n* = 381), tetracycline (TET: *n* = 717), and sulfamethoxazole/trimethoprim (SXT: *n* = 639). All disks were obtained from Becton Dickinson (USA), except for cefovecin disks (Oxoid, UK). The results were interpreted according to CLSI criteria [18] (for AMC, GEN, MAR, ENR, TET, and SXT), the European Committee on Antimicrobial Susceptibility Testing recommendations [19] (for CXM, CTX, and NOR), and the standards of the Comité de l'Antibiogramme de la Société Française de Microbiologie, Antibiogramme vétérinaire [20] (for AMX, NEO, and STR). As official breakpoints for cefovecin have not yet been available, we applied interpretive criteria proposed by Šeol et al. [21].

### 2.3. Analysis of Trends in Antimicrobial Resistance

The analysis was performed for antimicrobials for which the results for at least 30 isolates were available from each year of the study. Trends in resistance were analyzed for the following antimicrobials: amoxicillin (70–153 isolates each year), amoxicillin/clavulanic acid (71–152 isolates each year), tetracycline (71–149 isolates each year), trimethoprim/sulfamethoxazole (70–133 isolates each year), gentamicin (70–151 isolates each year), and streptomycin and neomycin (71–151 isolates each year for both); cefovecin and cefotaxime were combined into the extended-spectrum cephalosporins (ESCs: 30–143 isolates each year), whereas norfloxacin, enrofloxacin, and marbofloxacin were combined into fluoroquinolones (65–152 isolates each year).

### 2.4. Multidrug Resistance Analysis

Multidrug resistance analysis was carried out according to the definition proposed by Magiorakos et al. [22]. The isolate resistant to at least one antimicrobial in at least three categories was identified as multidrug-resistant. The analysis was performed for seven antimicrobial categories (representative antimicrobials used in the analysis given in brackets): penicillin (amoxicillin), penicillin with *β*-lactamase inhibitor (amoxicillin/clavulanic acid), ESCs (cefotaxime, cefovecin), aminoglycosides (gentamicin), fluoroquinolones (norfloxacin, enrofloxacin, and marbofloxacin), tetracyclines (tetracycline), and folate pathway inhibitors (sulfamethoxazole/trimethoprim). The analysis included 485 isolates for which complete records on the resistance to the aforementioned seven antimicrobial categories were available.

### 2.5. Statistical Analyses

Changes in the antimicrobial resistance during the seven-year period of the study were analyzed using chi-square test for trends [23]. Maximum likelihood chi-square test was used to compare prevalence of MDR between canine and feline* E. coli* isolates, between hemolytic and nonhemolytic* E. coli* isolates, and between* E. coli* isolates causing different types of infection. Modified Tukey's HSD post hoc test was used in the case of multiple comparisons [23]. For all statistical estimations, 95% confidence intervals (95% CI) were computed using the Wilson Score method [24]. A two-tailed *P* value below 0.05 was considered to indicate statistical significance. Statistical analyses and graphs were performed in Excel 2007 (Microsoft) and Statistica 10 (StatSoft Inc.).

## 3. Results

### 3.1. Antimicrobial Susceptibility

**Figure 1:**
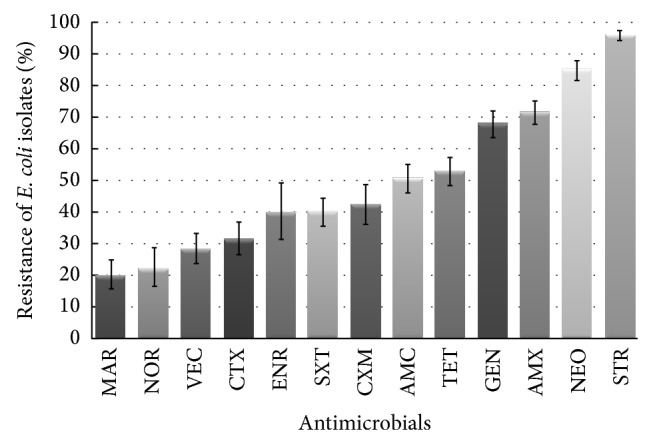
Antimicrobial resistance of* E. coli* isolates recovered from diseased dogs and cats in Poland between January 2007 and December 2013. AMC: amoxicillin/clavulanic acid, AMX: amoxicillin, CTX: cefotaxime, VEC: cefovecin, CXM: cefuroxime, ENR: enrofloxacin, GEN: gentamicin, MAR: marbofloxacin, NEO: neomycin, NOR: norfloxacin, STR: streptomycin, SXT: sulfamethoxazole/trimethoprim, and TET: tetracycline.

Results of antimicrobial susceptibility testing of* E. coli* isolates from clinical samples recovered from dogs and cats are presented in Figure 1 (intermediate isolates were included in group of the resistant isolates). The bacteria showed the lowest resistance to marbofloxacin, norfloxacin, cefovecin, and cefotaxime, with the frequency of 19.7%, 21.9%, 28.2%, and 31.4%, respectively. A higher percentage of resistance was found to enrofloxacin (39.3%), sulfamethoxazole/trimethoprim (39.9%), cefuroxime (41.2%), amoxicillin/clavulanic acid (52.1%), and tetracycline (53.0%). The highest percentage of resistance was observed to streptomycin (96.4%), neomycin (85.1%), amoxicillin (70.2%), and gentamicin (68.1%). There was no statistically significant difference in the susceptibility patterns to any of the antimicrobials between the* E. coli* isolates from dogs and cats or those from various types of infections.

### 3.2. Trends in Antimicrobial Resistance

**Figure 2:**
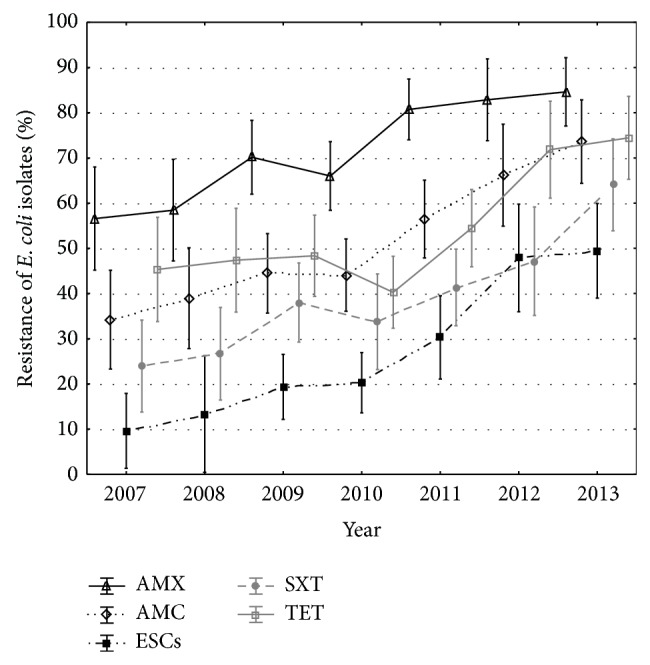
Increase in the frequency of resistance of canine and feline* E. coli* isolates in the seven-year period (2007–2013). AMC: amoxicillin/clavulanic acid, AMX: amoxicillin, ESCs: the extended-spectrum cephalosporins, SXT: sulfamethoxazole/trimethoprim, and TET: tetracycline.

During the seven-year period of the study, a statistically significant increase in frequency of resistant* E. coli* was observed in seven antimicrobials: amoxicillin (from 56.6% in 2007 to 73.6% in 2013; *P* < 0.001), amoxicillin/clavulanic acid (from 34.2% to 84.6%; *P* < 0.001), ESCs (from 9.6% to 49.5%; *P* < 0.001), tetracycline (from 45.3% to 74.4%; *P* < 0.001), sulfamethoxazole/trimethoprim (from 23.9% to 64.0%; *P* < 0.001), gentamicin (from 52.0% to 87.9%; *P* < 0.001), and neomycin (from 78.9% to 98.9%; *P* = 0.007) (Figure 2). No change of resistance level was observed for fluoroquinolones (*P* = 0.701) and streptomycin (*P* = 0.059); however, the resistance to the latter antibiotic had been over 90% for the entire study.

### 3.3. Multidrug Resistance

**Figure 3:**
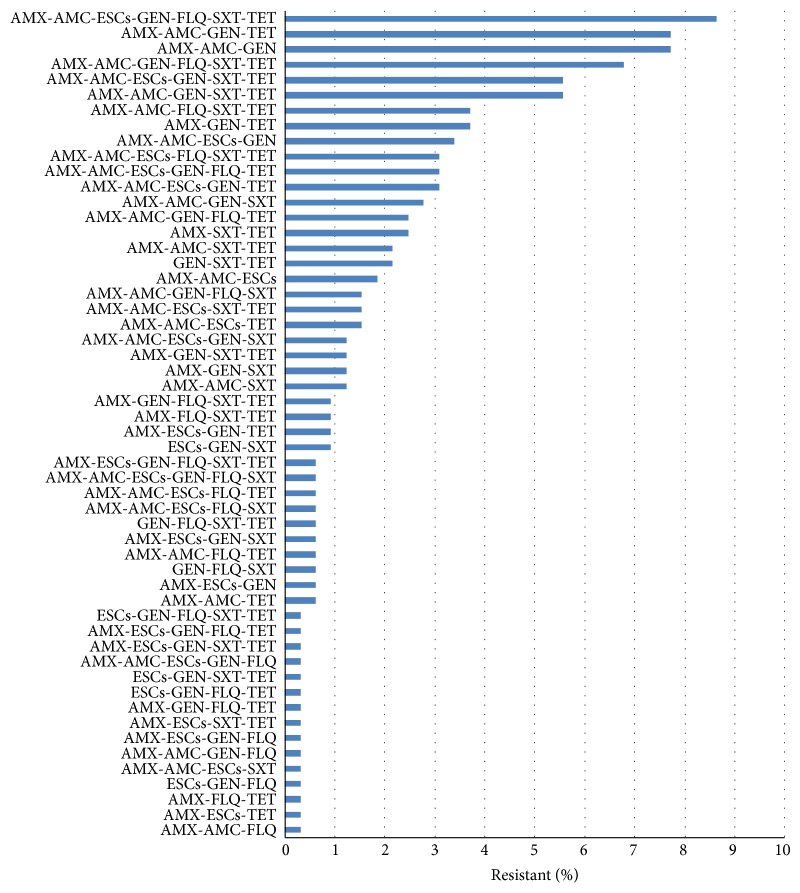
Multidrug resistance phenotypes in canine and feline MDR* E. coli* isolates (*n* = 324). AMC: amoxicillin/clavulanic acid (the representative of penicillin with *β*-lactamase inhibitor), AMX: amoxicillin (the representative of penicillin), ESCs: the extended-spectrum cephalosporins, FLQ: fluoroquinolones, GEN: gentamicin (the representative of aminoglycosides), SXT: sulfamethoxazole/trimethoprim (the representative of folate pathway inhibitors), and TET: tetracycline (the representative of tetracyclines).

**Figure 4:**
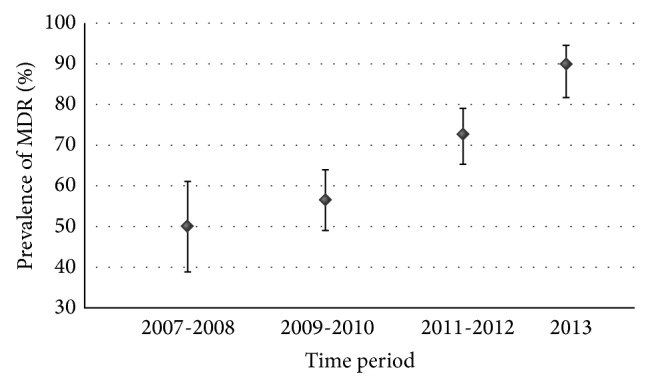
Change of the prevalence of multidrug resistance among* E. coli* isolated from diseased dogs and cats in Poland between January 2007 and December 2013.

**Figure 5:**
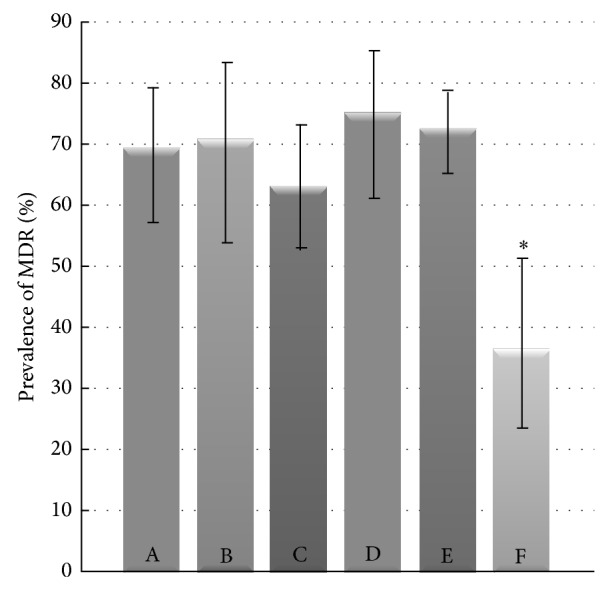
Percentage of MDR* E. coli* isolated from various types of infections in dogs and cats. A stands for skin and ear infections (*n* = 65), B soft tissue infections (*n* = 34), C gastrointestinal tract infections (*n* = 119), D respiratory tract infections (*n* = 48), E urinary tract infections (*n* = 178), and F reproductive tract infections (*n* = 41). ^*^ *P* < 0.01.

The analysis was performed for 485 isolates from which 261 were collected from dogs and 224 from cats (53.8% and 46.7%, resp.); 277 were hemolytic and 208 nonhemolytic (57.1% and 42.9%, resp.); 366 were isolated from extraintestinal and 119 from intestinal infections (75.5% and 24.5%, resp.). Forty-eight isolates were collected in 2007, 26 in 2008, 105 in 2009, 63 in 2010, 92 in 2011, 62 in 2012, and 89 in 2013. The distribution of resistance to multiple antimicrobial categories is shown in Table 2. Only few isolates were susceptible to all or to none of seven antimicrobial categories tested. The resistance to between one and five antimicrobial categories was distributed evenly. The multidrug resistance phenotype, defined as resistance to three or more antimicrobial categories, was detected in 324* E. coli* isolates (66.8%; 95% CI: 62.5% to 70.8%). MDR isolates were represented by 54 phenotypes (Figure 3). The percentage of MDR isolates increased from 50.0% (95% CI: 38.9% to 61.1%) in 2007-2008 to 89.9% (95% CI: 81.9% to 94.6%) in 2013 (*P* < 0.001) (Figure 4). There was no difference in the frequency of MDR* E. coli* isolation from dogs and cats (64.8% versus 69.2%; *P* = 0.299). No difference in the prevalence of multidrug resistance was also observed between isolates causing intestinal and extraintestinal infections (63.0% versus 68.0%; *P* = 0.317); however, the prevalence was lower in reproductive tract infections (36.6%) than in any other type of infection (63.0% to 75.0%) (*P* = 0.001) (Figure 5). Nonhemolytic* E. coli* isolates were multidrug resistant more often than hemolytic isolates (72.1% versus 62.8%; *P* = 0.031).

## 4. Discussion

The acquisition of antimicrobial resistance in bacteria has been noted for many years and has become one of the most important therapeutic problems in human and veterinary medicine. In this study we analyzed the occurrence of antimicrobial resistance in* E. coli* isolated from diseased dogs and cats.

The low percentage of isolates resistant to fluoroquinolones was found. These antimicrobials are often used to treat dogs and cats [25–27]. We evaluated the susceptibility of bacteria to fluoroquinolones of the second (norfloxacin, enrofloxacin) and third (marbofloxacin) generation. The percentage of strains resistant to enrofloxacin was slightly higher than that noted to marbofloxacin, which may be due to prolonged and more intensive usage of this antimicrobial agent in pets. Unexpectedly, bearing in mind their common use in veterinary practice, no increase in frequency of resistance to fluoroquinolones was observed during the seven-year period of this study. Thus, these antimicrobials are likely to remain the most effective therapeutics for* E. coli* infections in dogs and cats in Poland. However, a progressive decrease of fluoroquinolones' efficacy against Enterobacteriaceae has been observed in Australia [28]. Moreover, recently novel mutations in topoisomerase and* qepA* genes and plasmid-mediated fluoroquinolone resistance determinants were detected in ESBL-producing* E. coli* of canine and feline origin [29]. Regarding these newly recognized mechanisms of resistance, attentive monitoring of fluoroquinolone resistance seems to be especially important.

Most of* E. coli* isolates were susceptible to ESCs, such as cefotaxime and cefovecin belonging to the third cephalosporin generation. The resistance to cephalosporins of earlier generations was relatively high (above 40% to cefuroxime) and this result is consistent with those of previous studies on canine and feline* E. coli* isolates from different countries [30, 31]. However, increasing frequency of resistance to ESCs was observed between 2007 and 2013, implying dissemination of* E. coli* isolates producing cephalosporinases or ESBLs among companion animals in Poland, but this should be confirmed by further investigations.

A high frequency of resistance to aminopenicillins has been reported among clinical* E. coli* isolates of different origin worldwide [32]. Our results for amoxicillin confirmed this observation. The increasing resistance to amoxicillin/clavulanic acid in the study period is especially alarming, because this antimicrobial is very often used by veterinarians [1, 27]. This resistance can be associated, among others, with the activity of class C *β*-lactamases or inhibitor-resistant TEM *β*-lactamases [33, 34].

A low level of susceptibility to tetracyclines and sulfamethoxazole/trimethoprim has been previously demonstrated in animal* E. coli* isolates [3, 14, 26, 30]. The results of our study indicate that the resistance to these antimicrobials is currently increasing and they should be used only if the susceptibility of the bacteria is confirmed by* in vitro* study.

The resistance to the remaining antimicrobials tested against canine and feline* E. coli* isolates was very high, especially for aminoglycosides, such as gentamicin, streptomycin, and neomycin. A high resistance to these antibiotics has also been reported for* E. coli* isolates from food-producing animals in Europe [3, 35]. The most important mechanism of aminoglycoside resistance is the activity of different aminoglycoside-modifying enzymes. In* E. coli*, the gentamicin resistance is most commonly mediated by AAC(3)-I, AAC(3)-II, AAC(3)-IV, and ANT(2′′)-I enzymes, and the increasing frequency of genes encoding these enzymes in the clinical isolates of human and animal origin has been reported [36, 37]. Our results indicate that* E. coli* strains occurring in pets could be a substantial reservoir of the aminoglycoside resistance genes.

Multidrug resistance of bacterial pathogens isolated from food-producing and companion animals has become an emerging problem. The prevalence of multidrug resistance in* E. coli* causing infections in dogs and cats in Poland is alarming. Two-thirds (66.8%) of the isolates tested were classified as MDR. Most studies showed lower percentage of MDR* E. coli* in companion animals, for example, 43.3% in Japan [38] and 28.9% in USA [6]. The discrepancy between our and others' results could have two sources. Firstly, there is no universal definition of multidrug resistance and criteria applied for classification of isolates as MDR might differ. In the majority of studies, multidrug resistance was defined as resistance to two or more antimicrobial classes, all *β*-lactams being included in one class. However, this does not agree with the guidance of Schwarz et al. [39], who suggested counting separately the resistance to particular subgroups of *β*-lactams. In our study, following the multidrug resistance definition for Enterobacteriaceae proposed by Magiorakos et al. [22], *β*-lactams were divided into three different categories, which are an equivalent of three classes. Secondly, our study was carried out on samples delivered by veterinarians. In Poland microbiological tests are rarely ordered on the first visit. Like everywhere in Europe [40], empirical therapy with standard antibiotic is usually commenced and further laboratory investigation is performed only if this antibiotic, and not rarely one or two alternatives, prove ineffective. Therefore it is reasonable to assume that our study population included companion animals with* E. coli* infections resistant to initial treatment, so-called clinically problematic cases. It is known that an antimicrobial selective pressure is the most important factor stimulating acquisition of resistance and dissemination of MDR pathogens.* E. coli* isolates from healthy dogs and cats, not exposed to antimicrobials, show low level of resistance [41]. Therefore, the actual occurrence of MDR* E. coli* in the whole Polish population of dogs and cats is probably lower than what is reported in this study. However, our results imply that therapeutic problems with* E. coli* infections are considerably associated with the multidrug resistance of bacteria.

In our study, the occurrence of MDR* E. coli* was at the same level in dogs and cats, and also the frequency of MDR* E. coli* isolation from gastrointestinal and extraintestinal infections was similar. The prevalence of multidrug resistance in the isolates from reproductive tract infections was significantly lower. The MDR phenotypes were found significantly more often among the nonhaemolytic* E. coli* isolates. A higher level of resistance in nonhaemolytic* E. coli* was also noted by Pedersen et al. [26]. Clinical MDR* E. coli* isolates appear to be significantly less virulent and frequently belong to phylogenetic group A, B1, or D [16, 42, 43]. On the other hand, Platell et al. [10] observed the MDR phenotype in virulent* E. coli* isolates of sequence type 131, which belong to phylogroup B2 and are emerging among companion animals.

In the case of MDR bacteria, evaluation of their susceptibility to less commonly used antimicrobials is essential. On the other hand, the range of antimicrobials used in companion animal practice should depend not only on individual medical indications but also on the general epidemiological consideration of emerging bacterial resistance in both veterinary and human medicine. Veterinary guidelines for the responsible use of antimicrobials have been developed by different organizations [44]. Unfortunately in many countries those recommendations are not applied commonly in veterinary practice.

In conclusion, the patterns of antimicrobial resistance of* E. coli* isolates causing various types of infections in dogs and cats in Poland were similar. The results showed also that companion animals in Poland are an important reservoir of MDR* E. coli* strains. However, further investigations of antimicrobial resistance mechanisms in canine and feline* E. coli* population in Poland are required. Given the increasing resistance of bacteria to drugs commonly used in small animal veterinary practice, it seems crucial that treatment of bacterial infections should be conducted according to antibiogram results. Only prudent, reasonable, and appropriate use of antimicrobials can minimize the emergence of MDR bacteria. Therefore, the continuous monitoring of canine and feline* E. coli* antimicrobial susceptibility and the application of recommendations for antimicrobial use in small animal practice have clinical relevance and public health importance.

## Figures and Tables

**Table 1:** Origin of *E. coli* isolates from diseased animals.

Infection of	Clinical specimen	Number (%) of isolates		
		Dogs	Cats	Total
Urinary tract	Urine	122 (16.7)	122 (16.7)	244 (33.4)
Gastrointestinal tract	Fecal swab	90 (12.3)	108 (14.8)	198 (27.1)
Reproductive tract	Vaginal swab	35 (4.8)	36 (4.9)	71 (9.7)
Respiratory tract	Nasal, pharyngeal, tracheal, or conjunctival swab	50 (6.8)	17 (2.3)	67 (9.1)
Soft tissue	Internal organs, pleural, or peritoneal fluid	30 (4.1)	31 (4.3)	61 (8.4)
Skin or ear	Skin swab	30 (4.1)	6 (0.8)	36 (4.9)
	Ear canal swab	27 (3.8)	5 (0.7)	32 (4.5)
	Wound or abscess swab	17 (2.3)	4 (0.6)	21 (2.9)

Total		401 (54.9)	329 (45.1)	730 (100)

**Table 2:** Analysis of multidrug resistance among *E. coli* isolates (*n* = 485).

Number of antimicrobial categories to which isolate is resistant	Percentage (number) of resistant isolates	Classification
0	4.7% (23)	Non-MDR *E. coli* *n* = 161 (33.2%)
1	14.7% (71)	
2	13.8% (67)	

3	16.3% (79)	MDR *E. coli* *n* = 324 (66.8%)
4	16.5% (80)	
5	15.0% (73)	
6	13.2% (64)	
7	5.8% (28)	

## References

[1] Guardabassi L., Schwarz S., Lloyd D. H. (2004). Pet animals as reservoirs of antimicrobial-resistant bacteria. *Journal of Antimicrobial Chemotherapy*.

[2] Garcia-Migura L., Hendriksen R. S., Fraile L., Aarestrup F. M. (2014). Antimicrobial resistance of zoonotic and commensal bacteria in Europe: the missing link between consumption and resistance in veterinary medicine. *Veterinary Microbiology*.

[3] Bywater R., Deluyker H., Deroover E. (2004). A European survey of antimicrobial susceptibility among zoonotic and commensal bacteria isolated from food-producing animals. *Journal of Antimicrobial Chemotherapy*.

[4] Krutkiewicz A., Sałamaszyńska-Guz A., Rzewuska M., Klimuszko D., Binek M. (2009). Resistance to antimicrobial agents of *Campylobacter* spp. strains isolated from animals in Poland. *Polish Journal of Veterinary Sciences*.

[5] Wieler L. H., Ewers C., Guenther S., Walther B., Lübke-Becker A. (2011). Methicillin-resistant staphylococci (MRS) and extended-spectrum beta-lactamases (ESBL)-producing *Enterobacteriaceae* in companion animals: nosocomial infections as one reason for the rising prevalence of these potential zoonotic pathogens in clinical samples. *International Journal of Medical Microbiology*.

[6] Shaheen B. W., Boothe D. M., Oyarzabal O. A., Smaha T. (2010). Antimicrobial resistance profiles and clonal relatedness of canine and feline *Escherichia coli* pathogens expressing multidrug resistance in the United States. *Journal of Veterinary Internal Medicine*.

[7] Ho P. L., Chow K. H., Lai E. L. (2011). Extensive dissemination of CTX-M-producing *Escherichia coli* with multidrug resistance to “critically important” antibiotics among food animals in Hong Kong, 2008–10. *Journal of Antimicrobial Chemotherapy*.

[8] Cunha M. P. V., de Oliveira M. G. X., de Oliveira M. C. V. (2014). Virulence profiles, phylogenetic background, and antibiotic resistance of *Escherichia coli* isolated from Turkeys with Airsacculitis. *The Scientific World Journal*.

[9] Ewers C., Grobbel M., Stamm I. (2010). Emergence of human pandemic O25:H4-ST131 CTX-M-15 extended-spectrum-*β*-lactamase-producing *Escherichia coli* among companion animals. *Journal of Antimicrobial Chemotherapy*.

[10] Platell J. L., Cobbold R. N., Johnson J. R. (2011). Commonality among fluoroquinolone-resistant sequence type ST131 extraintestinal *Escherichia coli* isolates from humans and companion animals in Australia. *Antimicrobial Agents and Chemotherapy*.

[11] Beutin L. (1999). *Escherichia coli* as a pathogen in dogs and cats. *Veterinary Research*.

[12] Dierikx C. M., van Duijkeren E., Schoormans A. H. W. (2012). Occurrence and characteristics of extended-spectrum-*β*-lactamase- and AmpC-producing clinical isolates derived from companion animals and horses. *Journal of Antimicrobial Chemotherapy*.

[13] Huber H., Zweifel C., Wittenbrink M. M., Stephan R. (2013). ESBL-producing uropathogenic *Escherichia coli* isolated from dogs and cats in Switzerland. *Veterinary Microbiology*.

[14] Kroemer S., El Garch F., Galland D., Petit J.-L., Woehrle F., Boulouis H.-J. (2014). Antibiotic susceptibility of bacteria isolated from infections in cats and dogs throughout Europe (2002–2009). *Comparative Immunology, Microbiology & Infectious Diseases*.

[15] Sidjabat H. E., Townsend K. M., Lorentzen M. (2006). Emergence and spread of two distinct clonal groups of multidrug-resistant *Escherichia coli* in a veterinary teaching hospital in Australia. *Journal of Medical Microbiology*.

[16] Osugui L., Pestana de Castro A. F., Iovine R., Irino K., Carvalho V. M. (2014). Virulence genotypes, antibiotic resistance and the phylogenetic background of extraintestinal pathogenic *Escherichia coli* isolated from urinary tract infections of dogs and cats in Brazil. *Veterinary Microbiology*.

[17] CLSI (2013). *Performance Standards for Antimicrobial Disk and Dilution Susceptibility Tests for Bacteria Isolated From Animals*.

[18] CLSI (2013). *Performance Standards for Antimicrobial Disk and Dilution Susceptibility Tests for Bacteria Isolated From Animals*.

[19] EUCAST (2013). *Breakpoint Tables for Interpretation of MICs and Zone Diameters. Version 3.1, 2013*.

[20] CA-SFM (2010). *Comité de l'Antibiogramme de la Société Française de Microbiologie, Groupe de travail: antibiogramme vétérinaire, recommandations*.

[21] Šeol B., Matanović K., Mekić S., Starešina V. (2011). In vitro activity of cefovecin, extended-spectrum cephalosporin, against 284 clinical isolates collected from cats and dogs in Croatia. *Veterinarski Arhiv*.

[22] Magiorakos A.-P., Srinivasan A., Carey R. B. (2012). Multidrug-resistant, extensively drug-resistant and pandrug-resistant bacteria: an international expert proposal for interim standard definitions for acquired resistance. *Clinical Microbiology and Infection*.

[23] Zar J. H. (2010). *Biostatistical Analysis*.

[24] Altman D. G., Machin D., Bryant T. N., Gardner M. J. (2000). *Statistics with Confidence*.

[25] Lloyd D. H. (2007). Reservoirs of antimicrobial resistance in pet animals. *Clinical Infectious Diseases*.

[26] Pedersen K., Jensen H., Finster K., Jensen V. F., Heuer O. E. (2007). Occurrence of antimicrobial resistance in bacteria from diagnostic samples from dogs. *Journal of Antimicrobial Chemotherapy*.

[27] Murphy C. P., Reid-Smith R. J., Boerlin P. (2012). Out-patient antimicrobial drug use in dogs and cats for new disease events from community companion animal practices in Ontario. *The Canadian Veterinary Journal*.

[28] Gibson J. S., Cobbold R. N., Kyaw-Tanner M. T., Heisig P., Trott D. J. (2010). Fluoroquinolone resistance mechanisms in multidrug-resistant *Escherichia coli* isolated from extraintestinal infections in dogs. *Veterinary Microbiology*.

[29] Shaheen B. W., Nayak R., Foley S. L., Boothe D. M. (2013). Chromosomal and plasmid-mediated fluoroquinolone resistance mechanisms among broad-spectrum-cephalosporin-resistant *Escherichia coli* isolates recovered from companion animals in the USA. *Journal of Antimicrobial Chemotherapy*.

[30] Ball K. R., Rubin J. E., Chirino-Trejo M., Dowling P. M. (2008). Antimicrobial resistance and prevalence of canine uropathogens at the Western College of Veterinary Medicine Veterinary Teaching Hospital, 2002–2007. *The Canadian Veterinary Journal*.

[31] Shaheen B. W., Nayak R., Foley S. L. (2011). Molecular characterization of resistance to extended-spectrum cephalosporins in clinical *Escherichia coli* isolates from companion animals in the United States. *Antimicrobial Agents and Chemotherapy*.

[32] Belmar-Liberato R., Gonzalez-Canga A., Tamame-Martin P., Escribano-Salazar M. (2011). Amoxicillin and amoxicillin-clavulanic acid resistance in veterinary medicine—the situation in Europe: a review. *Veterinarni Medicina*.

[33] Philippon A., Arlet G., Jacoby G. A. (2002). Plasmid-determined AmpC-type *β*-lactamases. *Antimicrobial Agents and Chemotherapy*.

[34] Thomson K. S. (2010). Extended-spectrum-*β*-lactamase, AmpC, and carbapenemase issues. *Journal of Clinical Microbiology*.

[35] Hendriksen R. S., Mevius D. J., Schroeter A. (2008). Prevalence of antimicrobial resistance among bacterial pathogens isolated from cattle in different European countries: 2002–2004. *Acta Veterinaria Scandinavica*.

[36] Ho P.-L., Wong R. C., Lo S. W., Chow K.-H., Wong S. S., Que T.-L. (2010). Genetic identity of aminoglycoside-resistance genes in Escherichia coli isolates from human and animal sources. *Journal of Medical Microbiology*.

[37] Soleimani N., Aganj M., Ali L., Shokoohizadeh L., Sakinc T. (2014). Frequency distribution of genes encoding aminoglycoside modifying enzymes in uropathogenic *E. coli* isolated from Iranian hospital. *BMC Research Notes*.

[38] Harada K., Niina A., Nakai Y., Kataoka Y., Takahashi T. (2012). Prevalence of antimicrobial resistance in relation to virulence genes and phylogenetic origins among urogenital *Escherichia coli* isolates from dogs and cats in Japan. *The American Journal of Veterinary Research*.

[39] Schwarz S., Silley P., Simjee S. (2010). Editorial: assessing the antimicrobial susceptibility of bacteria obtained from animals. *Journal of Antimicrobial Chemotherapy*.

[40] de Briyne N., Atkinson J., Pokludová L., Borriello S. P., Price S. (2013). Factors influencing antibiotic prescribing habits and use of sensitivity testing amongst veterinarians in Europe. *Veterinary Record*.

[41] Costa D., Poeta P., Sáenz Y. (2008). Prevalence of antimicrobial resistance and resistance genes in faecal *Escherichia coli* isolates recovered from healthy pets. *Veterinary Microbiology*.

[42] Johnson J. R., Kuskowski M. A., Gajewski A., Sahm D. F., Karlowsky J. A. (2004). Virulence characteristics and phylogenetic background of multidrug-resistant and antimicrobial-susceptible clinical isolates of *Escherichia coli* from across the United States, 2000-2001. *The Journal of Infectious Diseases*.

[43] Wagner S., Gally D. L., Argyle S. A. (2014). Multidrug-resistant *Escherichia coli* from canine urinary tract infections tend to have commensal phylotypes, lower prevalence of virulence determinants and ampC-replicons. *Veterinary Microbiology*.

[44] Teale C. J., Moulin G. (2012). Prudent use guidelines: a review of existing veterinary guidelines. *Revue Scientifique et Technique*.

